# Determining unmet need: clinical relevance of suspected neurodivergence in first-episode psychosis

**DOI:** 10.1192/bjb.2024.64

**Published:** 2025-12

**Authors:** Nikola Nikolić, Catherine Sculthorpe, Jessica Stock, Dan Stevens, Jessica Eccles

**Affiliations:** 1 Early Intervention in Psychosis Service, Sussex Partnership NHS Foundation Trust, Worthing, UK; 2 Advanced Clinical Practice and Non-Medical Prescribing Department, London South Bank University, UK; 3 Department of Clinical Neuroscience, Brighton and Sussex Medical School, UK; 4 Neurodevelopmental Service, Sussex Partnership NHS Foundation Trust, Worthing, UK

**Keywords:** Autism spectrum disorders, attention-deficit hyperactivity disorders, neurodevelopmental disorders, first-episode psychosis, early intervention in psychosis

## Abstract

**Aims and method:**

We explored the prevalence of autism and attention-deficit hyperactivity disorder in first-episode psychosis. Through service evaluation involving 509 individuals, detailed analyses were conducted on neurodevelopmental traits and patterns of service utilisation.

**Results:**

Prevalence of neurodivergence in first-episode psychosis was 37.7%. Neurodivergent individuals used urgent mental health services more frequently (Mann–Whitney *U* = 25925, *Z* = −2.832, *P* = 0.005) and had longer hospital stays (Mann–Whitney *U* = 22816, *Z* = −4.886, *P* ≤ 0.001) than non-neurodivergent people. Neurodivergent people spend more than twice as long in mental health hospitals at a time than the non-neurodivergent people (Mann–Whitney *U* = 22 909.5, *Z* = −4.826, *P* ≤ 0.001). Mediation analysis underscored indirect impact of neurodivergence on hospital stay durations through age at onset of psychosis and use of emergency services.

**Clinical implications:**

Prevalence of neurodevelopmental conditions in first-episode psychosis is underestimated. Neurodivergent individuals show increased utilisation of mental health services and experience psychosis earlier. Early assessment is crucial for optimising psychosis management and improving mental health outcomes.

Over the past decade, there has been a growing interest in describing difficulties associated with the diagnostic overshadowing and assessment of neurodevelopmental conditions and psychosis. This has been an issue in early intervention in psychosis services (EIPS) nationally, as delayed misidentification of either of these may have a direct effect on the individual in meeting their support needs and designing their care package.

The increased co-occurrence of multiple neurodevelopmental conditions has become more prominent, such that up to half of individuals with autism have attention-deficit hyperactivity disorder (ADHD) characteristics, and two-thirds of individuals with ADHD have autistic characteristics.^1,2^ Although this common co-occurrence has been recognised in children, there is a paucity of research in adults.^3^ To our knowledge, the meta-analysis by Rong et al is the latest evidence that indicates that 40.2% of people with autism have experienced ADHD in their lifetime.^4^ A recent study by Underwood et al estimates that adults with autism are 10.74 times more likely to have an ADHD diagnosis than the general population.^5^

Treise et al^6^ sought to explore the unmet needs of co-occurring autism in psychosis. They propose a prevalence of 9% in individuals experiencing first-episode psychosis (FEP). This is significantly higher than the previously suggested prevalence of 3.6% in FEP, or the 2018 pooled estimate of diagnosed autism prevalence for people of all ages in England (1.57%). Strikingly, in Wales, the estimated overall prevalence of psychosis in autism is 18.3%.^5,7,8^

Over the past decade, data has emerged about prevalence of ADHD in psychotic disorders. Evidence from an English population survey suggests prevalence of suspected diagnosis of 0.53% in adults.^9^ Remarkably, a cohort of individuals with schizophrenia in London, demonstrated presence of ADHD symptoms through symptom screening in 23% of adults.^10^ The only study of individuals with a diagnosis of FEP known to us, reports that 15% of adults with FEP have a diagnosis of ADHD.^11^

To date, there is little evidence that examines both autism and ADHD in FEP. This is important, as ADHD is associated with earlier age at onset of FEP.^11^ Additionally, in individuals with autism and ADHD, ADHD significantly predicts conversion of prodromal symptoms of psychosis to FEP. So far, evidence is sparse in clarifying which clinical features of ADHD specifically pose the greatest risk. It also remains unclear to what extent ADHD treatment, i.e. dopaminergic agents, affect the risk of developing psychosis.^12–14^

Delays in treatment of psychosis (i.e. prolonged duration of untreated psychosis (DUP)), may negatively affect recovery, as they present an increased risk of relapse and result in poorer outcomes.^15^ Unmet support needs in neurodevelopmental conditions, such as autism and ADHD, may have detrimental effects on quality of life, including mental health and social difficulties, especially in young people.^16^ Remarkably, in October 2023, of 2035 patients admitted to in-patient mental health units in England, 44% of people were diagnosed with autism and an additional 22% were people with autism and an intellectual disability.^17^

## Aim

Our service seeks to promote a neurodiversity-affirmative approach, which aims to destigmatise ill health for neurodivergent individuals,^18,19^ and enable all individuals to improve their quality of life through access to the right support at the right time. To support this, we undertook a service-wide evaluation to establish prevalence of confirmed and suspected neurodivergent traits. A further aim was to establish whether presence of neurodivergent traits was associated with any difference in healthcare utilisation.

## Method

This service evaluation was set in the EIPS at Sussex Partnership NHS Foundation Trust (SPFT). This EIPS follows a full model of care, delivering all National Institute for Health and Care Excellence guidance and quality standards.^20^ The entire active case-load was audited in April 2023. Data were extracted from the electronic care records by PowerBI through Microsoft 365 for Windows (Microsoft, Redmond, USA; https://www.microsoft.com/en-us/power-platform/products/power-bi), and a representative sample (*n* = 120) was cross-referenced with the electronic care record to ensure data accuracy. A total of 519 records were identified. Individuals with a diagnosis of ‘at risk mental state’ (*n* = 7), otherwise known as clinically high risk, were excluded. There were two duplicates and one deceased individual, who were also excluded. This left a total of 509 individuals with suspected or confirmed FEP, aged 14–65 years at the point of referral to the service.

Each individual's record was screened in detail for the following:

- Confirmed diagnosis and/or suspected traits of neurodevelopmental conditions;
- Psychosis treatment pathway;
- DUP and age at onset of first psychotic symptom;
- Substance use status and risk associated with it;
- Number and length of each historic episode of care with any of the following services: mental health liaison team, crisis resolution home treatment, police street triage and emergency mental health crisis support (we will refer to these as emergency mental health services hereafter);
- Number and length of each historic admission to any mental health in-patient unit, including psychiatric intensive care units.

Out of the entire data-set, the missing data were within the following variables: DUP (*n* = 189, 37.1%), alcohol intake (*n* = 273, 53.6%) and amphetamine use (*n* = 455, 89.4%). Suspected neurodevelopmental conditions were identified either by clinicians’ direct observation and/or in some cases by the use of screening tools, such as the Ritvo Autism Asperger Diagnostic Scale–Revised, Autism Spectrum Quotient, Wender Utah Rating Scale, Adult ADHD Self-Report Scale and Camouflaging Autistic Traits Questionnaire.

All data were anonymised, stored securely in digital format only and handled in keeping with the principles of data protection. All statistical analyses were conducted with Statistical Package for the Social Sciences (SPSS; version 29 for Windows). Descriptive data were obtained, and independent-sample *t*-tests were used to examine the relationship between means for normally distributed data. Non-normally distributed data were subject to Mann–Whitney *U* analysis. Chi-squared statistics were used to test the relationship between categorical variables.

To explore potential associations between neurodivergence and the above listed variables, mediation analyses were performed. An estimation of indirect effects was performed with the PROCESS macro v3.5 for SPSS by Hayes.^21^ The 95% bootstrapped confidence interval for the indirect effect is based on 5000 samples, and is considered significant if the bootstrapped confidence intervals do not cross zero. We used two models: model 1, the predictor variable was neurodivergence, the outcome variable was total length of stay in a mental health hospital and the mediator was the age at which the first psychotic symptom was experienced; model 2, the predictor variable was neurodivergence, the outcome variable was total length of stay in a mental health hospital and the mediator was the number of episodes provided by an emergency mental health service (i.e. mental health liaison team, crisis resolution home treatment, police street triage and emergency mental health crisis support).

This project was classified and registered as a service evaluation via the SPFT Quality Improvement Team. It did not require ethical approval.^22^

## Results

A summary of the demographic data can be seen in Table 1.

**Table 1:** Demographic characteristics

Demographic question	Description	Frequency	Percentage
Age group (years)	14–17	15	2.9
	18–25	172	33.8
	26–40	190	37.3
	41–65	131	25.7
	>65	1	0.2
Gender	Male	318	62.5
	Female	188	36.9
	Non-binary	2	0.4
	Not specified	1	0.2
Ethnicity	BAME^a^	53	10.4
	Mixed/other	35	6.9
	Not known/not stated	117	23.0
	White	304	59.7
Neurodivergence	Not neurodivergent	317	62.3
	Confirmed ADHD	27	5.3
	Confirmed autism	37	7.3
	Confirmed neurodivergence^b^	53	10.4
	Suspected ADHD	63	12.4
	Suspected autism	114	22.4
	Any neurodivergence (suspected and/or confirmed)	192	37.7
Case-load pathway	Confirmed FEP	422	82.9
	Suspected FEP	87	17.1

BAME, Black, Asian and minority ethnic; ADHD, attention-deficit hyperactivity disorder; FEP, first-episode psychosis.

a. Combines Asian, Asian British, Black, Black British and any other minority ethnicity.

b. This includes confirmed diagnosis of any neurodivergence, including combination of more than one, i.e. autism only, ADHD only, both autism and ADHD.

### Primary analysis

The focus of analysis was to compare the use of SPFT services (emergency services and in-patient ward stays) across the three categories of neurodivergence groups against the non-neurodivergent group. These included any confirmed neurodivergence, any suspected neurodivergence, and combined confirmed and suspected neurodivergence. The Shapiro–Wilk test of normality was used on all of the variables to assess the assumption of normality. None of the variables in this analysis were normally distributed (all *P*-values >0.5, d.f. 119, significance at <0.001).

When we compared the use of emergency/crisis services including the mental health liaison team, crisis resolution home treatment, police street triage and emergency mental health crisis support, we found that the total combined days of service use was significantly higher in the confirmed neurodivergence group, at 44.1 *v.* 20.8 days (Mann–Whitney *U* = 9081.5, *Z* = −2.971, *P* = 0.003). Average length per episode was significantly longer in the confirmed neurodivergence group (Mann–Whitney *U* = 9584, *Z* = −2.477, *P* = 0.013). Average total length of all combined admissions per person to any in-patient mental health ward was significantly longer in the confirmed neurodivergence group, with means of 86.9 and 32.4 days, respectively (Mann–Whitney *U* = 9334, *Z* = −2.800, *P* = 0.005). All other comparisons were non-significant.

When we compared the suspected neurodivergence and non-neurodivergent group, we found that the total length of all combined admissions per person to any in-patient mental health ward was significantly longer in suspected neurodivergence group, with the means of 80.6 and 45.7, days respectively (Mann–Whitney *U* = 21 309, *Z* = −3.500, *P* < 0.001). The suspected neurodivergence group had a greater number of admissions to any in-patient mental health ward (Mann–Whitney *U* = 21 520, *Z* = −3.490, *P* < 0.001). On average, each admission in the suspected neurodivergence group was significantly longer than that in the non-neurodivergent group (Mann–Whitney *U* = 21 735, *Z* = −3.207, *P* < 0.001), and the age at which individuals experienced FEP in the suspected neurodivergence group was significantly lower than in the non-neurodivergent group (26.7 *v*. 33.9 years) (Mann–Whitney *U* = 2398.5, *Z* = −4.040, *P* < 0.001). All other comparisons were non-significant.

Finally, we compared the combined confirmed and suspected neurodivergence group, and the non-neurodivergent group against the same variables (see Table 2). DUP was the only variable with no significant difference between the two groups.

**Table 2:** Comparison of healthcare service utilisation between the combined confirmed and suspected neurodivergence group and non-neurodivergent group

Characteristic	Mean values, CSND group	Mean values, NND group	Mann–Whitney *U*	*Z*-value	*P-*value
Combined days of emergency service use	28.4	20.2	26 331.5	−2.557	0.011
Number of emergency episodes	3.8	3.1	25 925	−2.832	0.005
Average length of emergency episode (days)	8.1	5.7	27 216	−2.008	0.045
Combined days of admissions to in-patient mental health ward	93.4	32.8	22 816	−4.886	<0.001
Number of in-patient admissions	1.1	0.8	24 687.5	−3.834	<0.001
Average length of in-patient admission (days)	59.6	25.0	22 909.5	−4.826	<0.001
Age of experiencing FEP	26.0	34.9	2263.5	−5.186	<0.001

CSND, confirmed and suspected neurodivergence; NND, non-neurodivergent; FEP, first-episode psychosis.

### Secondary analysis

We examined the relationship between substance use and neurodivergence across the same three groups as described above. The relation between cocaine use and neurodivergence was significant (χ^2^(2, *N* = 509) = 8.534, *P* = 0.013), such that people in the suspected neurodivergence group were more likely to use cocaine than the people in the non-neurodivergent group. A similar significant relationship was observed in the combined confirmed and suspected neurodivergence group, such that these individuals were more likely to use cocaine than non-neurodivergent individuals (χ^2^(2, *N* = 509) = 11.036, *P* = 0.004). The relationship between 3,4- methylenedioxymethamphetamine (MDMA) use and neurodivergence was significant (χ^2^(2, *N* = 509) = 6.350, *P* = 0.043), meaning that individuals in the combined confirmed and suspected neurodivergence group were more likely to use MDMA than those in the non-neurodivergent group. We observed no significant relationships between alcohol, cannabis, amphetamines, legal highs and opiates among the individuals on the case-load.

### Mediation analyses

**Fig. 1:**
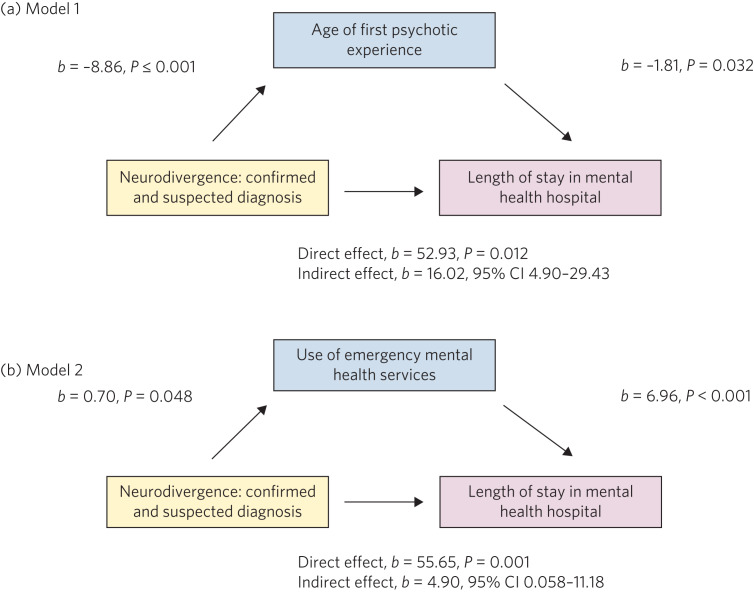
Predictor relationship between neurodivergence and length of stay in hospital is mediated by (a) age at which the first psychotic symptom was experienced and (b) number of emergency mental health services used by a person, such as mental health liaison team, crisis resolution home treatment, police street triage and emergency mental health crisis support.

Please see Fig. 1 for the indirect effect of neurodivergence in the specified models. In model 1, neurodivergence, confirmed and/or suspected, predicts total length of hospital stay through early age at onset of psychosis. In model 2, neurodivergence, confirmed and/or suspected, predicts total length of hospital stay via the utilisation of emergence mental health services.

## Discussion

Our study is the first study to explore the prevalence of both autism and ADHD, as the most co-occurring neurodevelopmental conditions, in individuals experiencing FEP. Our primary findings of prevalence of confirmed autism in FEP are similar to our colleagues Davidson et al and Treise et al, who both examined smaller cohort sizes of 197 and 210 individuals, respectively.^6,7^ On the contrary, our finding of confirmed diagnosis of ADHD (Table 1; 5.3%) is notably less than the most relevant study of ADHD in FEP (15%).^11^ It is important to recognise that the number of people who clinicians observed to have neurodevelopmental conditions traits was significantly higher, and in combination with those individuals already diagnosed with either or both neurodevelopmental conditions, the overall prevalence of neurodevelopmental conditions in FEP would in fact be 37.7% of the case-load.

We confirm that presence of neurodivergent traits has an effect on the use of both emergency mental health services and in-patient mental health units. We found that individuals with a diagnosed neurodevelopmental condition use emergency mental health services more than non-neurodivergent people, and when they are in mental health hospitals, their admissions are longer. When we compared individuals with suspected neurodivergence with non-neurodivergent individuals, not only were they also using emergency services more, but their admissions to in-patient mental health wards were also longer and they were experiencing their first psychotic symptoms at an earlier age. This last observation is in line with the findings by Rho et al.^11^

Once we combined individuals with confirmed and suspected neurodivergence, our results suggest that the neurodivergent individuals use emergency mental health services more often and for longer, each time, than non-neurodivergent people with a diagnosis of FEP. This group had more in-patient mental health admissions that were longer both in combined duration and individually, and they experienced their first psychotic experience nearly 9 years before their non-neurodivergent counterparts. Our mediation analysis confirms that the prolonged stays in mental health units are predicted by neurodivergence. Remarkably, this is indirectly affected through age at onset of psychosis, as well as the acuity associated with mental health challenges, resulting in the need for utilisation of emergency mental health services. Our findings strongly support the early intervention in psychosis model, and the critical need to early identify neurodivergence.

Future learning should be focused on a more systematic approach of establishing the prevalence of neurodevelopmental conditions in FEP, making use of established diagnostic tools with the least burden on the individual in view of time efficiency and accessibility, in addition to comprehensibility, clinician popularity and overall compliance with diagnostic standards such as the DSM or ICD. This is particularly relevant, as up to 99 different assessment tools are available in identification of autism alone.^23,24^ Appropriate and timely identification of neurodevelopmental conditions in FEP would allow for optimising management of psychosis and improving the trajectory of recovery from psychosis. Additionally, this would increase awareness of neurodevelopmental conditions in FEP, which would inevitably have a wider positive impact on other mental health challenges people with neurodivergence are faced with. Our healthcare system would greatly benefit from an approach that is truly inclusive, progressive and overall proactive. For this to be achieved, there must be a focus on enabling the workforce through investing in their education and training.

Our analysis of substance use indicated that cocaine and MDMA use is significantly more prevalent in neurodevelopmental conditions compared with non-neurodivergent individuals. In a meta-analysis and meta-regression analysis by van Emmerik-van Oortmerssen, cocaine dependence was associated with lower prevalence of ADHD when compared with alcohol, opioid or any other substance dependence.^25^ Although our results are not conclusive because of sample size and missing data, substance use remains an important element to be considered in neurodevelopmental conditions and psychosis, because of poorer outcomes associated with it.^26,27^

### Limitations

We recognise that we did not have a standardised system for screening suspected neurodevelopmental conditions, and that many factors are involved when comparing numbers of individuals with confirmed neurodevelopmental conditions as opposed to suspected, and this may vary from access to timely assessments and unequal distribution of resources, to individual clinician's skills as well as confidence and experience in working with neurodevelopmental conditions and psychosis. Our partial data on substance use underscores the need for further dedicated research to elucidate the implications of substance use within psychosis and neurodevelopmental conditions. Further, it is necessary to acknowledge that in the neurodivergent community, a number of individuals may not want to have a diagnosis of neurodevelopmental conditions for various reasons, but equally, that others are faced with challenges in a system that does not recognise self-diagnosis of neurodevelopmental conditions. This is further complicated by considerable service pressure in specialist neurodevelopmental condition services.

In conclusion, the prevalence of combined neurodevelopmental conditions, specifically autism and ADHD, on our case-load for FEP was 10.4%. It is highly possible that if all individuals with suspected traits of neurodevelopmental conditions were in fact confirmed as neurodivergent in accordance with the national guidelines, this number could be as high as 40%. Our data indicates that individuals with neurodivergence use mental health services more frequently and for longer periods of time than non-neurodivergent individuals. Individuals with neurodivergence are likely to be almost 9 years younger than non-neurodivergent persons at the time of experiencing their first symptom of psychosis. We conclude that there is a need for improving early intervention in psychosis services globally, to include early identification of all neurodevelopmental conditions and therefore help everyone live a meaningful, fulfilling life with the least amount of mental health challenges, regardless of their neurodevelopmental condition(s) status.

## Data Availability

The data that support the findings of this study are available from the corresponding author, N.N., on reasonable request.

## About the authors

**Nikola Nikolić** is a principal pharmacist and consultant clinical lead with the Early Intervention in Psychosis Service at Sussex Partnership NHS Foundation Trust, Worthing, UK; and a visiting fellow at the Advanced Clinical Practice and Non-Medical Prescribing Department of London South Bank University, UK. **Catherine Sculthorpe** is a professional lead for occupational therapy with the Early Intervention in Psychosis Service at Sussex Partnership NHS Foundation Trust, Worthing, UK. **Jessica Stock** is a clinical psychologist with the Early Intervention in Psychosis Service at Sussex Partnership NHS Foundation Trust, Worthing, UK. **Dan Stevens** is a clinical psychologist with the Early Intervention in Psychosis Service at Sussex Partnership NHS Foundation Trust, Worthing, UK. **Jessica Eccles** is a reader in brain–body medicine at the Department of Clinical Neuroscience of Brighton and Sussex Medical School, UK; and a liaison consulltant psychiatrist with the Neurodevelopmental Service at Sussex Partnership NHS Foundation Trust, Worthing, UK.
